# Pediatric Eosinophil-Predominant Collagenous Gastritis: From Conventional Therapy to Dupilumab: A Case Report

**DOI:** 10.3390/reports9030313

**Published:** 2026-09-15

**Authors:** Maria Rogalidou, Amalia Patereli, Kalliopi Stefanaki, Konstantina Dimakou, Eleanna Stasinou, Vasiliki-Maria Karagianni, Daphne Margoni, Alexandra Papadopoulou

**Affiliations:** 1 Division of Gastroenterology, Hepatology & Nutrition, 1st Department of Pediatrics, “Agia Sofia” Children’s Hospital, Medical School, National & Kapodistrian University of Athens, Thivon & Papadiamantopoulou Street, Goudis, 11527 Athens, Greece; 2 Pathology Department, “Agia Sofia” Children’s Hospital, 11527 Athens, Greece; 3 Department of Gastroenterology, “Agia Sofia” Children’s Hospital, 11527 Athens, Greece

**Keywords:** collagenous gastritis, dupilumab, iron deficiency anemia, case report, children

## Abstract

**Background and Clinical Significance:** Collagenous gastritis (CG) is a rare disorder characterized by subepithelial collagen deposition, typically presenting in children with iron deficiency anemia (IDA) and abdominal pain. Its pathogenesis remains unclear, and no standardized treatment exists. **Case Presentation:** A female patient developed IDA at 4.5 years of age, requiring repeated intravenous iron infusions, and recurrent epigastric pain. CG was diagnosed at 13.5 years, with marked gastric eosinophilia (peak 685 eosinophils/mm^2^) and subepithelial collagen thickening (103.6 μm). Extensive evaluation excluded alternative causes. Proton pump inhibitor (PPI) therapy failed to meaningfully improve symptoms, endoscopic findings, or histology. A 10-week course of oral systemic corticosteroids resolved abdominal pain and normalized hemoglobin and ferritin levels, but endoscopic abnormalities persisted, histologic improvement was limited, and pain recurred after withdrawal. Dupilumab was initiated as a steroid-sparing, histology-targeted therapy. Over 15 months, the patient remained asymptomatic, hemoglobin and ferritin levels remained normal without intravenous iron or routine oral iron supplementation, and endoscopic abnormalities improved although residual histologic abnormalities persisted. Dupilumab achieved a decrease in gastric eosinophil counts and subepithelial collagen thickness by 41% and 33%, respectively; steroids achieved decreases of 17% and 13%, respectively, and PPIs achieved decreases of 23% and 0.6%, respectively. **Conclusions:** Dupilumab was associated with sustained clinical and hematologic remission together with gradual endoscopic and histologic improvement in pediatric CG with prominent eosinophilic inflammation. To our knowledge, this is the first reported pediatric case of CG treated with dupilumab. These findings suggest that dupilumab may represent a promising steroid-sparing therapeutic option and support further evaluation in larger case series and controlled studies.

## 1. Introduction and Clinical Significance

Collagenous gastritis (CG) is a rare histopathological condition characterized by thickened subepithelial collagen bands in the gastric mucosa, typically exceeding 10 μm, accompanied by a mixed (lymphocytic- or eosinophilic-predominant) inflammatory infiltrate within the lamina propria [1,2]. Beinvogl et al. performed a retrospective study in 40 children and adolescents with CG and reported that 38.5% had increased lamina propria, and 15.3% had focal abnormal intraepithelial migration of eosinophils [1].

First described in a pediatric patient in 1989, subsequent pediatric cases have remained limited and are mainly reported as case reports and case series [2,3,4,5,6,7,8]. A Swedish population-based study estimated the incidence of childhood-onset CG at approximately 0.25 per 100,000 person-years [8].

In children and adolescents, the most common clinical manifestations are abdominal pain and iron deficiency anemia (IDA) [7]. Although disease involvement is usually confined to the stomach in pediatric cases, extension to the small intestine or colon has been reported, resulting in a broader clinical spectrum depending on disease distribution [3,4,5,6,7,8,9]. More extensive gastrointestinal (GI) involvement is generally associated with increased symptom severity and may necessitate targeted therapeutic strategies [9].

Histologically, CG is defined by dense subepithelial collagen deposition and predominantly lymphocytic or eosinophilic infiltrates in the lamina propria [2]. These changes may be associated with glandular atrophy and characteristic flattened or depressed mucosal appearance on endoscopy.

The pathogenesis of CG remains incompletely understood. The prevailing hypothesis proposes that collagen deposition represents a reparative response to prior mucosal injury, potentially triggered by inflammatory, autoimmune, infectious, or toxic factors in genetically predisposed individuals [1,2,3,4].

An alternative hypothesis suggests that microvascular abnormalities may increase vascular permeability, leading to protein leakage and secondary collagen accumulation [3]. The natural history and optimal management of CG remain unclear, as no randomized controlled trials have evaluated treatment strategies. While many patients experience symptomatic improvement over time, histological abnormalities often persist, and their long-term clinical significance is uncertain [7].

In adults, CG has been reported in association with eosinophilic gastrointestinal disorders; however, this overlap is rarely described in pediatric populations, with only isolated cases reported [9]. One pediatric case has described features overlapping with Eosinophilic gastritis [10].

In adults, treatments such as budesonide, proton pump inhibitors (PPIs), and dietary elimination have shown clinical benefit, although histological improvement in collagen deposition is uncommon, occurring in approximately 25% of cases [9]. Dupilumab is a monoclonal antibody targeting interleukin-4 (IL-4) and interleukin-13 (IL-13), key cytokines in type 2 (Th2)-mediated inflammation. It is approved for several allergic and inflammatory diseases, including atopic dermatitis, asthma, and chronic rhinosinusitis with nasal polyposis. Dupilumab is approved for the treatment of eosinophilic esophagitis (EoE) in children older than 1 year who weigh at least 15 kg, based on data from a prospective, randomized, placebo-controlled study [11]. More recently, it has also been evaluated in adolescents and adults with eosinophilic gastritis [12]. However, based on a literature search conducted while writing this manuscript, its use in pediatric collagenous gastritis has not been reported.

We report a case of collagenous gastritis with marked eosinophilic infiltration that achieved sustained clinical remission and showed endoscopic and histological improvement with dupilumab in combination with proton pump inhibitors, after prior treatment with proton pump inhibitors alone or combined with systemic steroids, and with no significant adverse effects other than transient mild peripheral eosinophilia.

To our knowledge, this is the first reported pediatric case of collagenous gastritis with an eosinophilic component treated with dupilumab. A PubMed search performed on August 30 2026, using the terms ‘collagenous gastritis’ AND ‘dupilumab’ AND ‘children’, identified no prior report of pediatric collagenous gastritis treated with dupilumab.

## 2. Case Presentation

We report the case of a female patient who first presented to our department at 12 years of age with severe IDA requiring repeated intravenous iron infusions, accompanied by mild to occasional moderate epigastric pain.

She had a history of IDA since the age of 4.5 years. Initial evaluation by a pediatric hematologist confirmed the diagnosis, and oral iron supplementation was initiated with an initially adequate hematologic response. Over time, hemoglobin levels ranged from 11.2 to 12.9 g/dL and hematocrit from 32.9% to 38.2%, while ferritin levels remained persistently low (17–21 ng/mL), consistent with ongoing iron depletion.

She was born at term with a normal birth weight and had normal growth throughout childhood. From her family history, her mother had Hashimoto’s thyroiditis, but there was no family history of allergies or atopy. The patient had no relevant comorbidities or history of intestinal parasitic infection. She was breastfed and introduced to complementary foods normally. She was not taking contraceptives or any medications other than those prescribed during the course of her illness.

At 11 years of age, she was hospitalized at a private hospital for severe anemia (Hb 7.5 g/dL, Ht 24.8%, ferritin 3 ng/mL) complicated by a syncopal episode. A comprehensive evaluation, including upper and lower gastrointestinal endoscopy, abdominal ultrasound, and magnetic resonance imaging, failed to identify a bleeding source. Upper endoscopy demonstrated mild gastritis. Gastric biopsies revealed rare Helicobacter pylori organisms; however, ^13^C urea breath test, PCR, and culture were all negative.

At 12 years of age, she was admitted to the Hematology Department of our hospital for a third IV iron infusion due to persistent iron deficiency anemia (Hb 9.2 g/dL, ferritin ~6 ng/mL), which increased her hemoglobin to 14.9 g/dL. Physical examination was unremarkable. The patient was hemodynamically stable, with normal growth parameters and no abdominal tenderness, organomegaly, or other clinically significant findings. Given her mother’s recent H. pylori gastritis, and although diagnostic criteria for active infection in the patient were not met according to the ESPGHAN/NASPGHAN guidelines [13], empirical eradication therapy was administered for 14 days with clarithromycin, amoxicillin, metronidazole, and esomeprazole, in case the persistent iron deficiency anemia was due to H. pylori organisms found in the gastric biopsy performed the previous year. Capsule endoscopy did not identify any small-bowel pathology.

**Figure 1:**
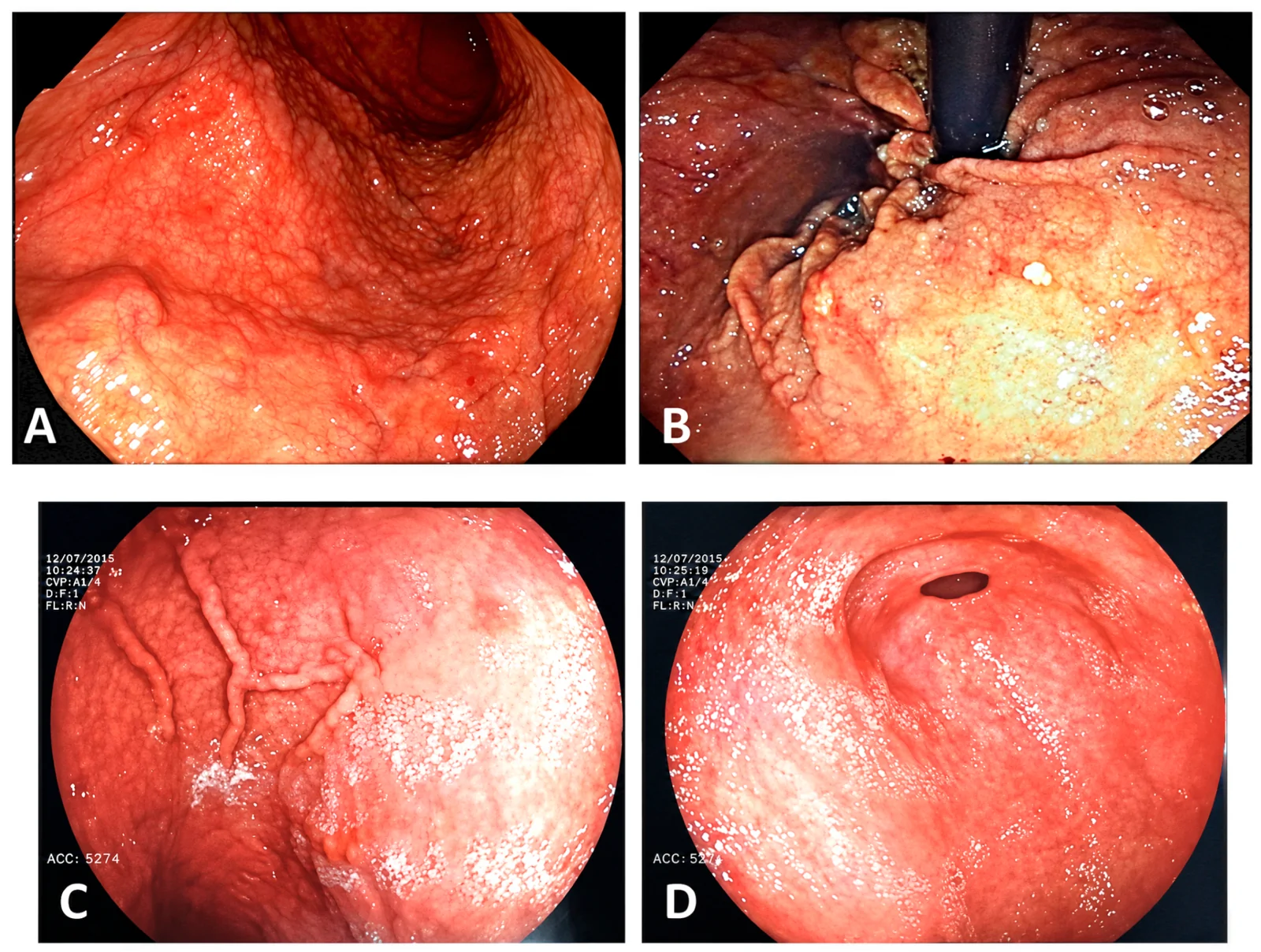
(**A**–**D**) Endoscopic abnormalities before and after treatment with dupilumab. (**A**) Initial endoscopy showing a nodular appearance of the gastric body and antrum. (**B**) After treatment with PPIs and systemic corticosteroids, showing a focal nodular appearance of the gastric body and antrum with multiple erosions. (**C**) Six months after the initiation of dupilumab, showing a focal nodular appearance of the gastric body with only one superficial erosion. (**D**) Fifteen months after the initiation of dupilumab, showing a focal nodular appearance of the gastric body without ulceration.

At 13 years 6 months of age, the patient was admitted again to our hospital for further evaluation because of persistent epigastric pain despite the administration of esomeprazole (1 mg/kg/day) for 3 months before the admission, and recurrent iron deficiency anemia. The physical examination was unremarkable, as in the previous examinations. Upper GI endoscopy revealed hemorrhagic spots, erosions, nodularity, erythema, and superficial ulcerations involving the gastric fundus and antrum (Figure 1). Colonoscopy and magnetic resonance enterography were unremarkable. For histological assessment of the biopsies, an Olympus BX43 microscope (field number 22 mm; high-power field size 0.238 mm^2^) was used, and peak eosinophil counts were expressed per high-power field (eos/HPF) and as eos/mm^2^. Histological examination of gastric biopsies showed chronic, focally erosive lymphonodular gastritis with marked eosinophilia (peak eosinophil count 163 eos/HPF, corresponding to 685 eos/mm^2^), as well as subepithelial hyalinization and collagen deposition, with a maximum subepithelial collagen thickness (CT) of 103.6 μm, representing a tenfold increase above 10 μm, which is the diagnostic cutoff for CG [1]. Histological assessment of the remaining biopsies from the upper GI tract showed mild esophagitis without eosinophils but with mild basal membrane hyperplasia, and mild duodenitis, with peak eosinophil counts of 41 eos/HPF, corresponding to 172 eos/mm^2^.

To determine the thickness of the subepithelial collagen deposits, histochemical staining with Masson’s trichrome was performed to confirm that these were indeed collagen deposits. The hematoxylin—eosin slides were then scanned sequentially using the Navigo Visia Imaging whole-slide scanner, and collagen thickness was measured using the built-in software at the point of greatest collagen thickness, as confirmed by Masson’s trichrome histochemical staining, and the thickness was measured at a magnification of x100, provided that the corresponding tissue section was adequately oriented. Testing for Helicobacter pylori (histology, culture, PCR and RUT (CLO) test) was negative. Treatment with esomeprazole 1 mg/kg was continued, along with systemic oral iron supplementation.

During follow-up visits, hemoglobin levels fluctuated between 11.5 and 12.3 g/dL, while ferritin remained persistently low (5–7.5 ng/mL, with a single value of 13.6 ng/mL). Evaluation for malabsorption and autoimmune disease was negative, including celiac serology (tTG IgA, EMA IgA), immunoglobulin levels, and thyroid autoantibodies. Stool calprotectin was mildly elevated (~100 μg/g; laboratory cutoff <50 μg/g), with no additional evidence of inflammatory bowel disease.

**Figure 2:**
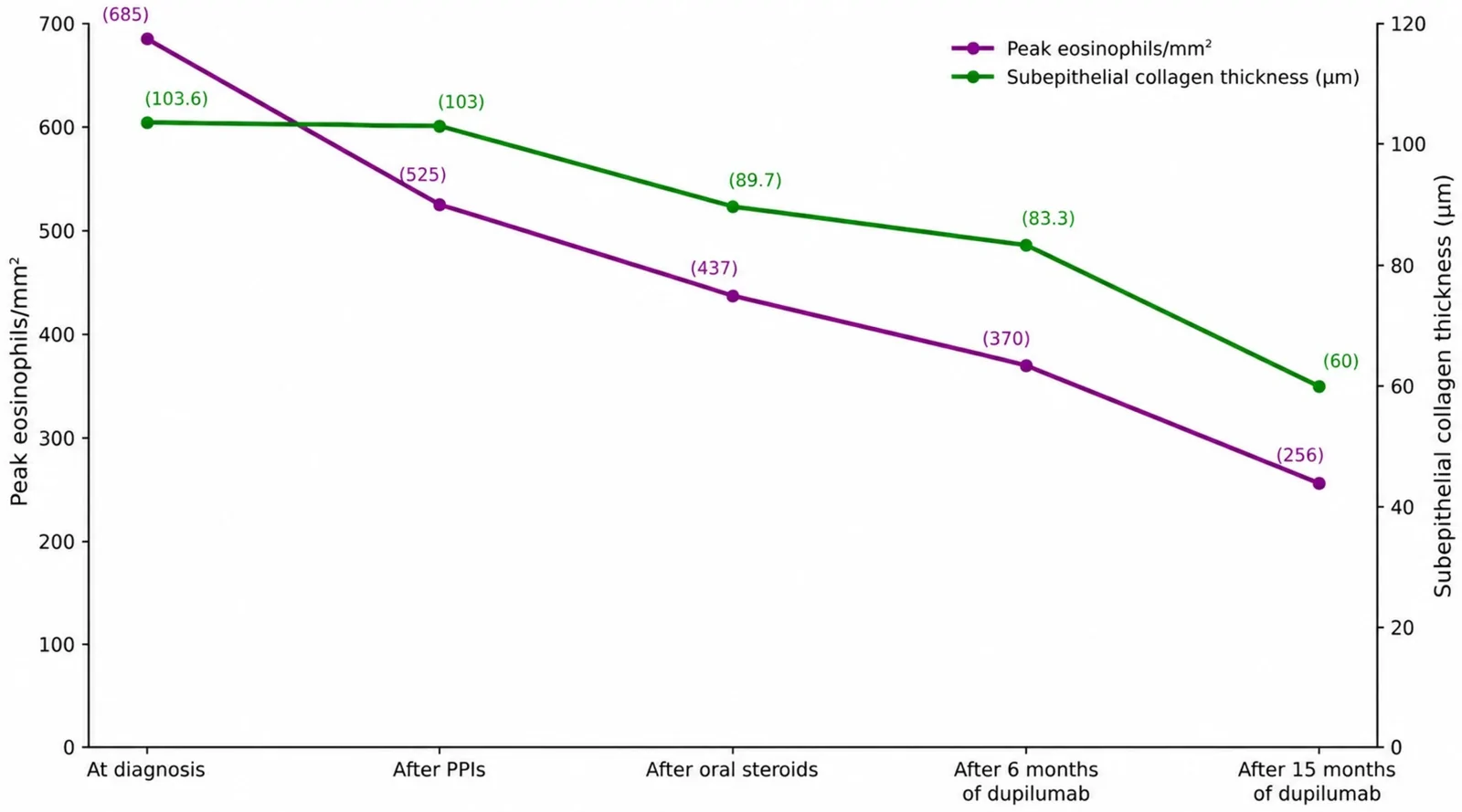
Subepithelial collagen thickness and eosinophilic infiltration of the gastric mucosa in response to different drug therapies. The x-axis is categorical, and each point is a single measurement with no replicates. Diagnostic threshold for collagenous gastritis: ≥10 μm [1]. Diagnostic Consensus threshold for childhood eosinophilic gastritis: peak ≥110 eosinophilic/mm^2^ [14].

At 13 years 11 months of age, the patient was readmitted to our hospital with abdominal pain and persistent iron deficiency anemia (Hb at admission 11.5, ferritin 5.2) despite systematic oral iron supplementation, requiring a fourth IV iron infusion. As with the previous admission, the physical examination was unremarkable. The patient was hemodynamically stable, with normal growth parameters and no abdominal tenderness, organomegaly, or other clinically significant findings. Upper gastrointestinal endoscopy showed a focal nodular appearance of the gastric body and antrum with multiple erosions, while the histology assessment showed slight histologic improvement in the gastric biopsies, with peak eosinophil counts of 125 eos/HPF (525 eos/mm^2^), representing a 23% reduction in eosinophil counts and almost no reduction (0.6%) in subepithelial CT (maximum CT 103 μm; Figure 2). Histologic examination of the rest of the GI segments revealed subepithelial fibrosis in esophageal biopsies, in the absence of eosinophilic mucosal infiltration, and mild duodenitis with peak eosinophil counts of 34 eos/HPF (143 eos/mm^2^).

At 14 years 1.5 months of age, after completion of a 10-week course of systemic oral steroids, starting with an initial dose of 40 mg/day for 2 weeks followed by a gradual taper and stop at 10 weeks, led to resolution of abdominal pain and a slight further improvement in the histologic abnormalities in gastric biopsies, with a 17% reduction in eosinophil counts (peak eosinophilc counts 104 eos/HPF corresponding to 437 eos/mm^2^) and a 13% reduction in collagen thickness (maximum CT 89.7 μm; Figure 2).

Unfortunately, 2 months after stopping steroids, abdominal pain recurred, although this was not accompanied by a fall in hemoglobin or ferritin levels while the patient was taking regular oral iron supplements. Physical examination was normal. Given the need for maintenance treatment, all available options were discussed with the patient and parents, including topical steroids, a milk elimination diet (due to the slight increase in total IgE although prick tests to cow’s milk, egg, soy, wheat, fish or nuts were all negative), and dupilumab. Because symptoms and iron status had improved during systemic corticosteroid therapy, but marked gastric eosinophilia and subepithelial collagen deposition persisted and corticosteroid dependence was considered undesirable, dupilumab, an IL-4/IL-13 pathway blocker, was started at the age of 14 years and 6 months, after informed consent and approval by the health authorities as a steroid-sparing, histologically targeted alternative. It was administered at a weight-based dose of 300 mg subcutaneously weekly, also intended to reverse esophageal fibrosis observed at the last upper gastrointestinal endoscopy.

During the first 6 months of therapy, the patient became completely asymptomatic, and hemoglobin and ferritin levels stabilized without the need for IV iron infusions or systemic oral iron supplementation; oral iron was administered only on days of menstrual bleeding.

At the end of 6 months of dupilumab treatment, at 15 years of age, endoscopic reevaluation showed macroscopic improvement, with only a single superficial erosion (Figure 1C), while histologic assessment of gastric biopsies showed a 15% decrease in eosinophil counts (peak 88 eos/HPF, corresponding to 370 eos/mm^2^) and a 7% decrease in collagen thickness (maximum CT 83.3 µm; Figure 2. Esophageal biopsies were normal with no eosinophils and no subepithelial fibrosis in the lamina propria but only mild basal membrane hyperplasia, while duodenal biopsies showed mild duodenitis with peak eosinophil counts of 23 eos/HPF corresponding to 97 eos/mm^2^.

**Figure 3:**
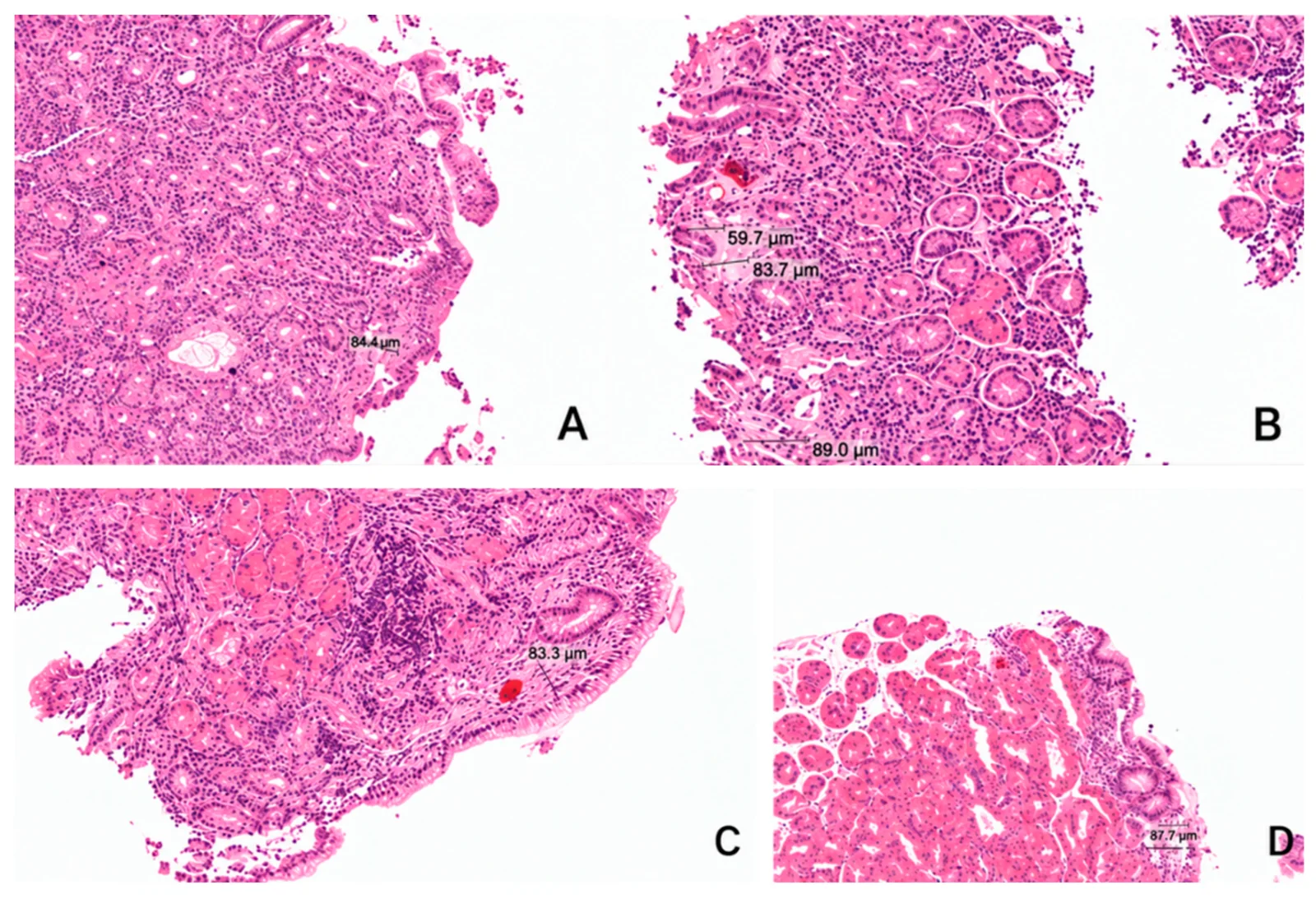
(**A**–**D**) Representative H&E-stained sections of gastric biopsies examined at magnification ×100 showing subepithelial collagen thickness following different drug therapies. (**A**) Gastric biopsies at diagnosis: The maximum subepithelial collagen thickness was 103.6 μm, confirming the diagnosis of collagenous gastritis. (**B**) Gastric biopsies after treatment with PPIs and systemic corticosteroids: The maximum subepithelial collagen thickness was 89.7 μm. (**C**) Gastric biopsies 6 months after initiation of dupilumab: The maximum subepithelial collagen thickness was 83.3 μm. (**D**) Gastric biopsies 15 months after initiation of dupilumab: The maximum subepithelial collagen thickness was 60 μm. The reported collagen data are descriptive, and the differences are within the range of sampling and measurement variability.

At 15 years 9 months of age, 15 months after treatment initiation, the patient remained asymptomatic and did not require iron infusions or systemic oral supplements to maintain hemoglobin and ferritin levels within the normal range (Table 1). Oral iron supplements were continued only on days of menstrual bleeding. The vitamin B12 level was 330 pg/mL. Inflammatory markers (CRP and ESR) were negative, and autoimmune screening remained unremarkable (negative ANA and anti-parietal cell antibodies). The serum gastrin level was elevated at 485 pg/mL (normal <250), which was attributed to PPI administration, which continued throughout; no baseline value before treatment initiation was available for comparison. Repeat endoscopy at that time demonstrated further macroscopic and histological improvement, with a 31% reduction in peak eosinophil counts (61 eos/HPF, corresponding to 256 eos/mm^2^) and a 28% reduction in collagen thickness (maximum CT 60 μm; Figure 2 and Figure 3).

Gastric biopsies were obtained from the antrum and corpus/fundus at each endoscopic evaluation. Given the inherent variability of endoscopic biopsy sampling, histological comparisons were made according to the documented gastric compartment rather than an identical anatomic location. Regarding other histological findings in the upper gastrointestinal tract, the pathologists reported mild esophagitis with no eosinophilic infiltration or subepithelial fibrosis in the lamina propria, as well as mild basal membrane hyperplasia. The pathologists also found chronic gastritis with mild activity, focal, mild hyalinized fibrosis (Figure 3), and mild duodenitis with peak eosinophil counts of 31 eos/HPF, corresponding to 130 eos/mm^2^. Adherence to weekly dupilumab was confirmed at follow-up visits by review of administered doses, and treatment was well tolerated. Improvement in histological abnormalities tended to be slightly greater with the combination of dupilumab and PPIs than with PPIs alone or PPIs combined with oral systemic steroids. A 41% reduction in tissue eosinophil counts and a 33% reduction in subepithelial CT were observed 15 months after initiation of dupilumab, compared with values at the start of treatment with this drug (Figure 2). However, because dupilumab therapy lasted six times longer than steroid therapy, we cannot draw firm conclusions about the superior efficacy of dupilumab compared with steroids. Moreover, the possibility that its combination with PPIs, which continued throughout the entire treatment period, contributed to the therapeutic effect of dupilumab cannot be ruled out. No adverse events or injection-site reactions were reported during the treatment period, apart from a temporary, slightly increased peripheral eosinophil count (Table 1). It should be noted, however, that slightly increased eosinophil counts in peripheral blood had already been observed earlier, including 2 months after discontinuation of oral systemic corticosteroid treatment (0.79 K/μL).

The CARE Clinical Timeline and the CARE check list have been added as supplementary documents (Supplementary Table S1 and Supplementary Table S2, respectively).

## 3. Patient Perspective

The patient described the prolonged diagnostic course, recurrent abdominal pain, repeated blood tests, hospital visits, and intravenous iron infusions as burdensome. She wished to avoid prolonged systemic corticosteroid treatment and preferred not to follow a restrictive elimination diet because of its potential impact on her daily activities and nutritional requirements. She and her parents therefore considered dupilumab an acceptable treatment option. During dupilumab therapy, she reported resolution of abdominal pain and was pleased that she no longer required intravenous iron infusions or routine oral iron supplementation. She considered the treatment well tolerated and was satisfied with her clinical improvement.

## 4. Discussion

CG often presents with nonspecific gastrointestinal symptoms and IDA, and its clinical features may overlap with those of eosinophilic gastrointestinal disorders, making differential diagnosis challenging [10,15]. Eosinophilic infiltration may coexist with CG, potentially influencing disease behavior and contributing to treatment resistance [10,15]. Although atopic conditions and food allergies have been reported in some patients [15], our patient had no personal or family history of atopy or food allergy. However, we should note the presence of maternal Hashimoto thyroiditis, consistent with the frequent familial predisposition to autoimmunity reported in childhood-onset CG (heredity for autoimmune disease in 47%), although that cohort documented no autoimmune comorbidity in the patients themselves [8].

The potential role of Helicobacter pylori in the pathogenesis of collagenous gastritis (CG) warrants consideration, as chronic mucosal inflammation may contribute to collagen deposition in selected cases [16]. Although rare H. pylori organisms were initially identified in gastric biopsies from our patient, ^13^C urea breath testing and culture were negative, and subsequent gastric biopsies, culture, and CLO testing were also negative. Therefore, H. pylori infection was not confirmed in our patient according to ESPGHAN/NASPGHAN guidelines [13]. Nevertheless, we decided to provide empiric treatment, which did not alter the disease course: abdominal pain persisted, and iron deficiency anemia continued, necessitating another IV iron infusion.

In eosinophilic gastritis, corticosteroid therapy was associated with a clinical response in 7 of 10 treated patients (70%), whereas 3 of 10 patients (30%) did not respond [17]. The authors could not identify clear histopathological predictors of response [17]. In our patient, systemic corticosteroids led to clinical, but not endoscopic, improvement, while histological improvement was moderate. Moreover, after discontinuation of steroids, abdominal pain recurred, but iron deficiency anemia did not.

Topically targeted budesonide and dietary elimination are potential therapeutic options for collagenous gastritis. A recent retrospective study [18] in 64 patients with CG (50 adults, 14 children) showed that 89% of patients had a clinical response to topically targeted budesonide (42% complete, 46% partial), and 88% had a histologic response (53% complete, 33% partial). However, the specific response rate to the drug among children with steroid-refractory disease was not reported.

In our case, the use of topical targeted budesonide, an elimination diet, or dupilumab was discussed with the patient and the parents. However, given the persistence of endoscopic abnormalities after systemic corticosteroid treatment, the modest reductions in eosinophilic inflammation and collagen thickness (17% and 13%, respectively) achieved after a 10-week course at adequate doses, and the limitations of prolonged steroid use, the patient and the parents declined topical steroids for maintenance. An elimination diet was also considered, but the potential nutritional burden of a restrictive regimen was viewed as a major challenge for our patient, a ballet performer with high nutritional and energy requirements. Thus, dupilumab was selected as an alternative targeted approach, without interpreting its use as evidence of superiority over topical budesonide or dietary therapy.

The management of collagenous gastritis remains largely empirical in both pediatric and adult patients, and no standard treatment has been established. Reported approaches include acid suppression, iron supplementation, corticosteroids, dietary interventions, and, in selected refractory cases, immunomodulatory therapies [1,4,5,9,15].

In our patient, prolonged PPI therapy and repeated iron supplementation, including intravenous iron, were insufficient to prevent persistent iron deficiency, abdominal pain, and ongoing endoscopic and histological abnormalities. Systemic corticosteroid therapy resolved symptoms and maintained hemoglobin and ferritin levels without further need for intravenous iron infusions, requiring only oral iron supplements; however, it did not achieve endoscopic or histological remission. Given the recurrence of abdominal pain after discontinuation of steroids and the persistent eosinophilic component of the disease, dupilumab was considered a rational therapeutic option to target type 2 inflammation. The subsequent achievement and maintenance of clinical remission; preservation of hemoglobin and ferritin levels within the normal range without the need for IV iron infusion or routine oral iron supplementation (except during menstrual bleeding), and the histologic improvement observed during dupilumab treatment suggest that eosinophilic/type 2 inflammation may have contributed to disease activity and may explain, at least in part, the observed response to dupilumab. On the other hand, the gradual histological improvement over time may also reflect the disease’s natural course; therefore, a causal relationship cannot be inferred. As the patient approaches adulthood, follow-up will transition from pediatric to adult gastroenterology care, with continued monitoring of symptoms, hematologic and iron status, and periodic endoscopic and histologic assessments according to the clinical course.

The management of CG remains challenging because of heterogeneous treatment responses and the frequent persistence of histologic abnormalities despite symptomatic improvement. The reported association between adult-onset CG and eosinophilic gastritis suggests that these entities may represent a disease spectrum in which chronic eosinophilic inflammation contributes to progressive subepithelial fibrosis and collagen deposition [9]. A similar overlap has also been described in pediatric patients [10,15]. Our case therefore represents an additional pediatric observation consistent with this hypothesis. However, an alternative interpretation has been proposed by Khurram et al., who classified collagenous gastritis as a potential secondary cause of gastric eosinophilia, suggesting that eosinophilic infiltration may result from underlying mucosal injury rather than represent a primary pathogenic mechanism [17]. Our findings cannot distinguish between these hypotheses. The improvement observed during dupilumab treatment may be compatible with a role for type 2 inflammation in CG. It should be noted, however, that a recent synthesis of 101 articles and approximately 730 histologically confirmed CG cases reported mixed Th1 and Th2 cytokine profiles in CG gastric tissue, together with α4β7-mediated mucosal homing, arguing against a purely type-2 model [19].

A recent pediatric case report described successful treatment with dupilumab in a 14-year-old patient with concurrent eosinophilic esophagitis and eosinophilic gastritis, resulting in clinical, endoscopic, and histologic improvement [20]. However, collagenous gastritis was not reported in that case. To the best of our knowledge, this is the first reported pediatric case of collagenous gastritis with prominent eosinophilic infiltration treated with dupilumab.

Dupilumab is a monoclonal antibody that targets interleukin-4 (IL-4) and interleukin-13 (IL-13), key cytokines in type 2 (Th2)-mediated inflammation. It is approved for several allergic and inflammatory diseases, including atopic dermatitis, asthma, chronic rhinosinusitis with nasal polyps, and, more recently, eosinophilic esophagitis (EoE), and is recommended for selected groups of patients with refractory or recurrent EoE [21]. Dupilumab has also been evaluated in eosinophilic gastrointestinal disorders, including eosinophilic gastritis, with emerging evidence of reduced tissue eosinophilia and improved endoscopic and histologic outcomes. These findings support consideration of IL-4/IL-13 pathway inhibition in selected patients with eosinophilic gastrointestinal disease, although evidence in collagenous gastritis remains limited [12,21].

In the phase 2 DEGAS randomized controlled trial, dupilumab significantly reduced gastric eosinophil counts in adults and adolescents with eosinophilic gastritis, achieving a 50% relative reduction from baseline compared with 4% with placebo at week 12, and was also associated with significant improvements in histologic and endoscopic outcomes [12]. Although clinical improvement based on the Eosinophilic Gastritis Symptom Questionnaire (EoG-SQ) did not reach statistical significance in that trial, observational studies have reported clinical benefit in non-eosinophilic esophagitis and eosinophilic gastrointestinal disorders [22]. An additional ongoing clinical trial (NCT05831176) is further evaluating the efficacy of dupilumab in adolescents and adults with eosinophilic gastritis with or without eosinophilic duodenitis.

Regarding the histological criteria for diagnosing non-EoE eosinophilic gastrointestinal diseases (non-EoE EGIDs), the criteria used in the DEGAS for adult patients and adolescents with eosinophilic gastritis differ from the histological criteria recommended for children and adolescents by the two leading societies in pediatric gastroenterology, hepatology, and nutrition: the European Society for Paediatric Gastroenterology, Hepatology and Nutrition (ESPGHAN) and the North American Society for Pediatric Gastroenterology, Hepatology and Nutrition (NASPGHAN) [14]. According to the joint ESPGHAN and NASPGHAN guidelines on non-EoE EGIDs [14], the diagnosis of non-EoE EGIDs must fulfill the following three criteria: a. Symptoms and/or signs of GI dysfunction; b. Dense eosinophilic infiltrates in mucosal or full-thickness biopsies above organ-specific threshold values (for eosinophilic gastritis, the consensus threshold for peak eosinophil counts was ≥110 eos/mm^2^); c. Absence of other diseases associated with GI mucosal eosinophilic inflammation [14]. Our patient had symptoms suggestive of GI impairment (recurrent epigastric pain and iron deficiency anemia) and met the histological criterion (peak eosinophil counts in gastric biopsies ≥110 eos/mm^2^), but not the third criterion of excluding a secondary cause of gastric eosinophilia, as a tenfold increase in subepithelial collagen thickness to 103.6 μm confirmed the diagnosis of CG [14]. Whether CG with predominantly eosinophilic inflammation falls within the spectrum of eosinophilic gastrointestinal disorders cannot be determined from a single case report and warrants further investigation.

With regard to the dose of dupilumab our patient received, there are currently no established dosing recommendations for dupilumab in pediatric non-EoE eosinophilic gastrointestinal diseases [14]. We followed the pediatric EoE label because of the concomitant fibrosis observed in the esophageal biopsy. We therefore used the regimen recommended for the child’s weight in EoE (300 mg once weekly), rather than the experimental regimen from the DEGAS study [12], and we did not administer a loading dose.

Treatment was continued in light of the clinical and objective improvements, with no significant side effects apart from a transient, mild peripheral eosinophilia, which had also been noted occasionally in the past, including 2 months after discontinuation of oral systemic steroids.

In our patient, dupilumab treatment was associated with sustained clinical, biochemical, endoscopic, and histological improvement. However, residual eosinophilic inflammation persisted after 15 months. It therefore remains uncertain whether longer treatment might lead to further histological improvement or complete remission, which will be assessed at subsequent endoscopic evaluations. Nevertheless, because this is a single case, these findings should be considered hypothesis-generating and require confirmation in larger case series and prospective randomized trials with placebo and other potentially effective drugs, such as topical steroids, which, in our case, were declined by the parents.

## 5. Conclusions

Collagenous gastritis is a rare and therapeutically challenging condition in pediatric patients, characterized by accompanying lymphocytic or eosinophilic infiltration. In this case, persistent iron deficiency and abdominal pain were associated with marked eosinophilic infiltration and significant collagen deposition. Targeted IL-4/IL-13 inhibition with dupilumab in our patient with CG and eosinophil-predominant inflammation was associated with sustained clinical and biochemical remission and histologic improvement, including reduced eosinophilic inflammation and subepithelial collagen deposition, although complete histologic remission was not achieved. Because the phases were sequential, unblinded, of unequal duration, and accompanied by continued acid suppression, these changes cannot be attributed to any single agent and no comparison of efficacy between treatments is possible. Dupilumab may be a therapeutic option for refractory or recurrent CG with an eosinophilic component when other, better-studied drugs, such as targeted topical budesonide, are unavailable, ineffective, or declined by the patient or parents. However, larger case series and prospective randomized trials are needed to confirm its safety and efficacy.

## Figures and Tables

**Table 1:** Clinical and hematological response to treatment.

	At Diagnosis *	During 6 Months of PPIs **	During 2.5 Months of Systemic Steroids ***	During the First 6 Months of Dupilumab ****	From 6–15 Months of Dupilumab *****
Abdominal pain	Yes	Yes	No	No	No
Lowest Hb (g/dL)	7.5	11.5	13	13	13.2
Lowest ferritin (ng/mL)	5.1	5.2	32.3	30.6	30.6
Absolute numbers of peripheral blood eosinophils (normal range: 0.02–0.55 K/μL)	0.34	0.55	0.44	0.70	0.08
IgE IU/mL (normal value <200)	163	-	-	-	-
Oral iron supplements	Yes	Yes	Yes	No #	No #
IV iron infusions	3	1	0	0	0
BMI kg/mm^2^ (CDC BMI-for-age z-scores) ^	17.9 (−0.44)	17.6 (−0.67)	17.4 (−0.80)	17.3 (−1.09)	18.06 (−0.90)

PPIs: proton pump inhibitors; * Age at diagnosis: 13 years and 6 months; ** Age at the end of treatment with PPIs as single drug: 13 years and 11 months; *** Age at the end of treatment with oral steroids plus PPIs: 14 years and 1.5 months; **** Age at 6 months of treatment with dupilumab plus PPIs: 15 years; ***** Age at 15 months of treatment with dupilumab plus PPIs: 15 years and 9 months; # Iron supplements were given only during menstrual bleeding. ^ BMI-for-age z-scores were calculated according to the CDC 2000 BMI-for-age Growth Charts using sex- and age-specific LMS parameters (L, Box–Cox power; M, median; S, coefficient of variation).

## Data Availability

The original contributions presented in this study are included in the article. Further inquiries can be directed to the corresponding author. The authors state their adherence to the CARE checklist in this manuscript.

## Supplementary Materials

The following supporting information can be downloaded at: https://www.mdpi.com/article/10.3390/reports9030313/s1, CARE Clinical Timeline (Supplementary Table S1); CARE checklist (Supplementary Table S2).

## Abbreviations

The following abbreviations are used in this manuscript:

CG	Collagenous gastritis
IDA	Iron deficiency anemia
GI	Gastrointestinal
eos/HPF	Eosinophils/per high-power field
CT	Collagen thickness
PPI	Proton pump inhibitors
PCR	Polymerase chain reaction
ESPGHAN	European Society for Paediatric Gastroenterology, Hepatology and Nutrition
HPF	High-power field
RUT (CLO)	Rapid urease test
tTG	Tissue transglutaminase
IgA	Immunoglobulin A
EMA	Endomysium antibodies
IL	Interleukin
CRP	C-reactive protein
ESR	Erythrocyte sedimentation rate
ANA	Antinuclear antibodies
EoE	Eosinophilic esophagitis
EoG-SQ	Eosinophilic Gastritis Symptom Questionnaire
NASPGHAN	North American Society for Pediatric Gastroenterology, Hepatology and Nutrition
