# Supplementary material for: Pediatric Eosinophil-Predominant Collagenous Gastritis: From Conventional Therapy to Dupilumab: A Case Report
Source: Reports (MDPI). 2026 Sep 15;9(3):313. doi: 10.3390/reports9030313 (PMC13611187; doi:10.3390/reports9030313)
Supplement: Supplementary file 1 [file reports-09-00313-s001.zip › Supplementary_Table_S1_CARE_Clinical_Timeline_Reports.pdf]

reports

MDPI

Supplementary Table S1. CARE Clinical Timeline

Pediatric Eosinophil-Predominant Collagenous Gastritis: From Conventional Therapy to Dupilumab. A Case Report

Supplementary material prepared from the final revised manuscript. Page and line references refer to the revised PDF with visible line numbering.

Chronological summary of clinical presentation, diagnostic evaluation, interventions, and outcomes.

Age / time point	Clinical presentation and key investigations	Treatment / intervention	Outcome / response	Manuscript location
**Age 4.5 years**	Iron deficiency anemia (IDA) diagnosed by a pediatric hematologist. Oral iron produced an initially adequate hematologic response; later Hb 11.2–12.9 g/dL, Hct 32.9–38.2%, ferritin persistently 17–21 ng/mL.	Oral iron supplementation initiated.	Persistent iron depletion over time.	Page 3, lines 107–111
**Age 11 years**	Hospitalized at a private hospital with severe anemia and syncope: Hb 7.5 g/dL, Hct 24.8%, ferritin 3 ng/mL. Upper/lower GI endoscopy, abdominal ultrasound and MRI did not identify a bleeding source. Upper endoscopy: mild gastritis. Gastric biopsies: rare H. pylori organisms; 13C urea breath test, PCR and culture negative.	Evaluation for bleeding source; iron replacement continued.	No bleeding source identified.	Page 3, lines 118–123
**Age 12 years**	Admitted to the Hematology Department for persistent IDA: Hb 9.2 g/dL, ferritin ~6 ng/mL. Physical examination unremarkable; normal growth parameters. Capsule endoscopy: no small-bowel pathology.	Third IV iron infusion (Hb increased to 14.9 g/dL). Empirical 14-day H. pylori eradication with clarithromycin, amoxicillin, metronidazole and esomeprazole.	IDA subsequently recurred; empiric eradication did not alter the later disease course.	Page 3, lines 124–134
**Age 13 years 6 months — diagnostic evaluation**	Persistent epigastric pain despite esomeprazole 1 mg/kg/day for the preceding 3 months and recurrent IDA. Upper GI endoscopy: hemorrhagic spots, erosions, nodularity, erythema and superficial ulcerations in fundus/antrum. Colonoscopy and MRE unremarkable. Gastric histology: chronic focally erosive lymphonodular gastritis, peak 163 eos/HPF (685 eos/mm²), maximum subepithelial collagen thickness (CT) 103.6 µm. Mild esophagitis without eosinophils; mild duodenitis, 41 eos/HPF (172 eos/mm²). H. pylori testing negative.	Esomeprazole 1 mg/kg/day continued; systemic oral iron supplementation continued.	Collagenous gastritis diagnosed with marked eosinophilic infiltration.	Pages 3–4, lines 135–162
**Follow-up during PPI-alone phase**	Hb 11.5–12.3 g/dL; ferritin 5–7.5 ng/mL (single value 13.6 ng/mL). Malabsorption/autoimmune evaluation negative; stool calprotectin mildly elevated (~100 µg/g).	Continued PPI and oral iron.	Persistent iron depletion and abdominal pain.	Page 4, lines 163–168
**Age 13 years 11 months — end of PPI-alone phase**	Readmitted with abdominal pain and persistent IDA: Hb 11.5 g/dL, ferritin 5.2 ng/mL. Endoscopy: focal nodularity of gastric body/antrum with multiple erosions. Gastric histology: 125 eos/HPF (525 eos/mm²), 23% reduction; maximum CT 103 µm, 0.6% reduction. Esophageal subepithelial fibrosis without eosinophilic mucosal infiltration; mild duodenitis, 34 eos/HPF (143 eos/mm²).	Fourth IV iron infusion; PPI continued; oral iron continued.	Only slight histologic improvement; symptoms/IDA persisted.	Page 4, lines 169–182
**Following 10-week systemic corticosteroid course; age 14 years 1.5 months**	After 10 weeks of oral systemic steroids: abdominal pain resolved. Gastric eosinophils 104 eos/HPF (437 eos/mm²), 17% reduction from the preceding assessment; maximum CT 89.7 µm, 13% reduction.	Oral systemic steroids: 40 mg/day for 2 weeks, followed by gradual taper and discontinuation at 10 weeks; PPI continued.	Clinical response with modest histologic improvement; no endoscopic remission.	Page 4, lines 183–188
**~2 months after stopping systemic steroids**	Abdominal pain recurred, without a fall in Hb or ferritin while taking regular oral iron. Physical examination normal. Peripheral eosinophils measured at 0.79 K/µL before dupilumab initiation.	Maintenance options discussed: topical steroids, milk elimination diet and dupilumab.	Need for steroid-sparing maintenance strategy identified.	Pages 4–6, lines 189–196 and 248–250
**Age 14 years 6 months — dupilumab initiated**	Persistent marked gastric eosinophilia and collagen deposition; prior esophageal fibrosis contributed to regimen selection.	Dupilumab 300 mg subcutaneously once weekly, weight-based pediatric EoE regimen; no loading dose. PPI continued. Informed consent and health-authority approval obtained.	Treatment initiated as a steroid-sparing, histologically targeted alternative.	Page 5, lines 197–202; page 11, lines 428–434
**First 6 months of dupilumab**	Patient became completely asymptomatic. Hb and ferritin stabilized without IV iron or routine systemic oral iron; oral iron only on days of menstrual bleeding.	Dupilumab 300 mg weekly + ongoing PPI; oral iron restricted to menstrual days.	Sustained clinical and hematologic improvement.	Page 5, lines 203–206

Age / time point	Clinical presentation and key investigations	Treatment / intervention	Outcome / response	Manuscript location
**Age 15 years — 6-month endoscopic reassessment**	Endoscopy: macroscopic improvement with a single superficial erosion. Gastric histology: 88 eos/HPF (370 eos/mm²), 15% decrease; CT reported as 83.3 µm in Figure 3 (7% decrease from 89.7 µm). Esophageal biopsies: no eosinophils or subepithelial fibrosis; mild basal membrane hyperplasia. Duodenum: 23 eos/HPF (97 eos/mm²).	Continue dupilumab weekly + PPI.	Clinical remission with endoscopic and histologic improvement.	Page 5, lines 207–214; pages 7–8, lines 272–277
**Age 15 years 9 months — 15 months of dupilumab**	Asymptomatic; no IV iron or routine systemic oral iron. Table 1: Hb 13.2 g/dL, ferritin 30.6 ng/mL, peripheral eosinophils 0.08 K/µL, BMI 18.06 kg/m² (z-score −0.90). Vitamin B12 330 pg/mL; CRP/ESR negative; ANA and anti-parietal cell antibodies negative; gastrin 485 pg/mL while on PPI. Endoscopy: further macroscopic improvement. Gastric histology: 61 eos/HPF (256 eos/mm²), 31% decrease from 6-month assessment; CT 60 µm, 28% decrease from 6-month assessment. Overall from dupilumab initiation: tissue eosinophils ↓41%, CT ↓33%.	Continue dupilumab 300 mg weekly + PPI; oral iron only during menstrual bleeding.	Sustained clinical/hematologic remission; continued endoscopic/histologic improvement, with residual histologic abnormalities. No injection-site reactions or other significant adverse events; transient mild peripheral eosinophilia noted during treatment.	Page 5, lines 215–247; Table 1 page 8, lines 282–289
**Ongoing follow-up**	Adherence to weekly dupilumab confirmed by review of administered doses. Plan for transition to adult gastroenterology with continued symptom, hematologic/iron, endoscopic and histologic monitoring according to clinical course.	Continue follow-up and treatment as clinically indicated.	Longer-term histologic outcome remains uncertain.	Page 5, lines 235–247; page 10, lines 361–364; page 11, lines 439–447
