# Supplementary material for: Pediatric Eosinophil-Predominant Collagenous Gastritis: From Conventional Therapy to Dupilumab: A Case Report
Source: Reports (MDPI). 2026 Sep 15;9(3):313. doi: 10.3390/reports9030313 (PMC13611187; doi:10.3390/reports9030313)
Supplement: Supplementary file 1 [file reports-09-00313-s001.zip › Supplementary_Table_S2_CARE_Checklist_Reports.pdf]

reports

Supplementary Table S2. CARE Checklist

Pediatric Eosinophil-Predominant Collagenous Gastritis: From Conventional Therapy to Dupilumab. A Case Report

Supplementary material prepared from the final revised manuscript. Page and line references refer to the revised PDF with visible line numbering.

CARE case-report checklist mapped to the revised manuscript.

Item	Topic	CARE checklist item	Reported?	Location in revised manuscript
1	**Title**	The words “case report” should be in the title along with the area of focus.	Yes	Page 1, lines 1–3
2	**Key Words**	2 to 5 key words that identify areas covered in this case report.	Yes	Page 1, line 42
3a	**Abstract — Introduction**	What is unique about this case and what does it add to the scientific literature?	Yes	Page 1, lines 16–19, 35–40
3b	**Abstract — Main symptoms/findings**	Main symptoms and/or important clinical findings.	Yes	Page 1, lines 20–23
3c	**Abstract — Diagnoses/interventions/outcomes**	Main diagnoses, therapeutic interventions, and outcomes.	Yes	Page 1, lines 23–34
3d	**Abstract — Conclusion**	Main take-away lesson(s) from this case.	Yes	Page 1, lines 34–40
4	**Introduction**	Brief background summary of the case with reference to the relevant medical literature.	Yes	Pages 2–3, lines 44–100
5a	**Patient Information — De-identified information**	De-identified demographic and other patient-specific information.	Yes	Page 3, lines 104–117
5b	**Patient Information — Main concerns/symptoms**	Main concerns and symptoms of the patient.	Yes	Page 3, lines 104–111; lines 135–140
5c	**Patient Information — Medical/family/psychosocial history**	Relevant medical, family, and psychosocial history, including relevant past interventions and outcomes.	Yes	Page 3, lines 107–134; page 8, lines 290–299
6	**Clinical Findings**	Relevant physical examination and other significant clinical findings.	Yes	Page 3, lines 124–128, 138–140; page 4, lines 172–174
7	**Timeline**	Historical and current information from this episode of care organized as a timeline.	Yes	Supplementary Table S1; chronology also in pages 3–6, lines 104–250
8a	**Diagnostic Assessment — Methods**	Diagnostic methods (e.g., physical examination, laboratory testing, imaging, questionnaires).	Yes	Pages 3–5, lines 118–182, 207–236
8b	**Diagnostic Assessment — Challenges**	Diagnostic challenges (e.g., access, financial or cultural factors).	Yes	No specific access/financial/cultural diagnostic barrier is described; diagnostic uncertainty and repeated negative evaluations are reported on pages 3–4, lines 118–168.
8c	**Diagnostic Assessment — Diagnosis/differential**	Diagnosis (including differential diagnoses considered).	Yes	Pages 3–4, lines 129–168; page 11, lines 408–427
8d	**Diagnostic Assessment — Prognostic characteristics**	Prognostic characteristics when applicable.	Yes	Residual histologic abnormalities and uncertainty about long-term histologic remission are discussed on page 11, lines 439–447.
9a	**Therapeutic Intervention — Types**	Types of intervention (pharmacologic, surgical, preventive, self-care).	Yes	Pages 3–5, lines 124–245
9b	**Therapeutic Intervention — Administration**	Administration of interventions (dose, strength, duration).	Yes	Page 3, lines 129–134; page 4, lines 160–162, 183–188; page 5, lines 197–202; page 11, lines 428–434
9c	**Therapeutic Intervention — Changes**	Changes in intervention with rationale.	Yes	Pages 4–5, lines 189–206; page 9, lines 332–341
10a	**Follow-up and Outcomes — Clinician/patient outcomes**	Clinician- and patient-assessed outcomes when appropriate.	Yes	Pages 4–5, lines 169–247; Patient Perspective page 8, lines 290–299
10b	**Follow-up and Outcomes — Follow-up tests**	Important follow-up diagnostic and other test results.	Yes	Pages 4–5, lines 169–247; Figures 1–3, pages 6–8

reports

MDPI

Item	Topic	CARE checklist item	Reported?	Location in revised manuscript
**10c**	**Follow-up and Outcomes — Adherence/tolerability**	Intervention adherence and tolerability.	Yes	Page 5, lines 235–247
**10d**	**Follow-up and Outcomes — Adverse/unanticipated events**	Adverse and unanticipated events.	Yes	Pages 5–6, lines 246–250; page 11, lines 435–438
**11a**	**Discussion — Strengths/limitations**	Strengths and limitations of the approach to this case.	Yes	Pages 9–12, especially page 10 lines 358–380 and page 11 lines 439–463
**11b**	**Discussion — Relevant literature**	Discussion of the relevant medical literature.	Yes	Pages 9–11, lines 301–438
**11c**	**Discussion — Rationale for conclusions**	Scientific rationale for conclusions, including possible causes.	Yes	Pages 9–11, lines 346–438
**11d**	**Discussion — Take-away lessons**	Primary take-away lessons from this case report.	Yes	Pages 11–12, lines 449–463
**12**	**Patient Perspective**	The patient should share their perspective or experience whenever possible.	Yes	Page 8, lines 290–299
**13**	**Informed Consent**	Was informed consent obtained?	Yes	Page 12, lines 482–484
